# Application of a New Type of Natural Calcined Bone Repair Material Combined with Concentrated Growth Factors in Bone Regeneration in Rabbit Critical-Sized Calvarial Defect

**DOI:** 10.1155/2020/8810747

**Published:** 2020-11-24

**Authors:** Xiaoyang Wang, Shuqing Tong, Shengyun Huang, Li Ma, Zhenxing Liu, Dongsheng Zhang

**Affiliations:** ^1^Department of Stomatology, Shandong Provincial Hospital, Cheeloo College of Medicine, Shandong University, Jinan, Shandong 250021, China; ^2^Department of Clinical Laboratory, Shandong Provincial Hospital, Shandong First Medical University, Jinan, Shandong 250021, China; ^3^Department of Stomatology, Shandong Provincial Hospital, Shandong First Medical University, Jinan, Shandong 250021, China; ^4^Department of Stomatology, Shandong Provincial Hospital, Cheeloo College of Medicine, Shandong University, Jinan, Shandong 250021, China

## Abstract

**Purpose:**

This study is aimed at investigating bone regeneration in critical-sized defects in rabbit calvarium using a novel nano- (n-) hydroxyapatite hybrid scaffold with concentrated growth factors (CGFs).

**Methods:**

Twenty-four male adult rabbits were chosen to establish a critical-sized bone defect model and randomly divided into two groups. Two defects of 15 mm diameter each were created in the parietal bone of each animal. Group A had n-hydroxyapatite hybrid scaffold placed in the experimental defect on the right, and the left defect was unfilled as blank. Group B had hydroxyapatite hybrid scaffold mixed with CGF placed in the right defect and CGF on the left. Six animals in each group were sacrificed after 6 and 12 weeks. Cone-beam computed tomography system scanning and hematoxylin and eosin (HE) staining were used to detect osteogenesis within the defects.

**Results:**

The treatment with n-hydroxyapatite hybrid scaffold along with CGF resulted in a significantly higher amount of new bone at 6 and 12 weeks compared to the treatment with CGF alone and the controls. No apparent inflammation and foreign body reaction were observed through HE staining.

**Conclusions:**

The new synthesized n-hydroxyapatite hybrid scaffold and CGF can be applied for bone defect regeneration to promote the process to a certain extent.

## 1. Introduction

The regeneration of bone defects in the maxillofacial skeleton is still challenging in the clinic, especially large defects in trauma, osteotomy surgery, oncologic resections, developmental anomalies, or socket preservation before implantation, in which defects need to be reconstructed [1, 2]. Autogenous bone grafts are the best material for defect reconstruction, as there is no rejection. However, problems remain as the grafts may be insufficient for large defects, which may lead to additional surgical trauma and significant resorption [3].

Hydroxyapatite bone substitutes have been widely used in the clinic over the years [4–6]. They can be obtained in many ways, making them immunologically harmless. They stimulate osteogenesis, incorporate into the defects, and provide a scaffold for the ingrowth of vessels and cells to facilitate new bone formation. Products vary in size or are coated by different elements, drugs, or growth factors and confirmed to promote osteogenesis [7–10]. However, problems remain in clinical use.

In this study, the nano- (n-) hydroxyapatite (HA) material was developed from Ostrea cucullate. Previous studies showed that n-hydroxyapatite is similar to human bone in the crystal structure and has no cytotoxicity. Thus, n-hydroxyapatite is expected to be effective in human bone regeneration.

Concentrated growth factor (CGF) [11], first developed by Sacco in 2006, was first obtained by centrifuging blood samples at alternating and controlled speeds. CGF contains more growth factors and harder fibrin than platelet-rich plasma (PRP) and platelet-rich fibrin (PRF) and is thought to promote osteogenesis in bone defects reconstruction.

This study was designed to verify bone regeneration in the critical-sized bone defect in the calvarium of rabbits using n-hydroxyapatite alone or combined with CGF. The expectation was to find a new kind of hydroxyapatite material that meets the requirements, which is more accessible, cheaper, and convenient.

## 2. Materials and Methods

The study design was approved by the ethical committee of the Medical College of Shandong University.

### 2.1. n-Hydroxyapatite and Collagenous Membrane

The n-hydroxyapatite (HA) was developed by Professor Zhang Qiqing's team in the Key Laboratory of Biomedical Engineering of Fujian province, College of Materials, Xiamen University. The n-HA was synthesized from oyster shell powder by hydrothermal reaction and was tested by X-ray diffraction (XRD), scanning electron microscopy (SEM), Fourier transform infrared spectroscopy (FTIR), energy dispersive spectroscopy (EDS), and MTT. The result showed that its crystal size and composition are similar to that of human bone. In vitro cytotoxicity test shows that the material has good biocompatibility, which is more conducive to the application of biomedical bone repair materials. The preparation process, crystal phase composition, morphology, chemical composition, and cytocompatibility test results were published in the Journal of Xiamen University (NATURAL SCIENCE) Vol. 49, No.5, Sep 2010. This research mainly focus on the animal experiment.

Collagenous membrane (GTR, produced by Bote Biotech company Ltd. Fujian, China).

### 2.2. CGF Preparation

**Figure 1:**
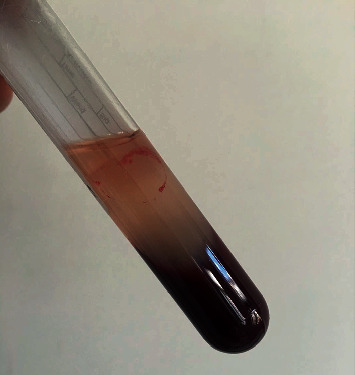
CGF was prepared from rabbit blood and then used for the animal experiments.

General anesthesia was induced by the intramuscular injection of 1 ml/kg of Pentobarbital at a dose of 3%. Preoperatively, 5 ml of intravenous blood was drawn from the posterolateral vein of the right hind leg. The blood samples were directly drawn into a sterile tube without anticoagulants and centrifuged (MedifugeTM, Silfradent Srl, Sofia, Italy) for approximately 13 min to produce CGF. After the centrifugation, samples were divided into three layers (Figure 1): the upper layer containing platelet-poor plasma, the middle yellow layer consisting of CGF, and the lower layer containing red blood cells. The middle layer was used for further analysis.

### 2.3. Animal Experiments

The critical-sized bone defects were introduced in the cranium of 24 New Zealand white rabbits, which were anesthetized via muscular injection with Pentobarbital at a dose of 3% and divided into two groups. One arc incision was made 1 cm behind the eyes, and the whole skin and periosteum were elevated to expose the skull. Two full-thickness critical-sized bone defects with a diameter of 15 mm were created on each side of the sagittal suture. The HA powder was placed in the right defect, and the left was kept empty in Group A. HA and CGF were placed in the right defect at a ratio of 1 : 1, and CGF alone was injected in the left in Group B. All defects were covered using a collagenous membrane. The skin was reset with interrupted suture. The time of evaluation for all groups was 6 and 12 weeks after the surgical procedure. Six animals in each group were sacrificed at these times.

### 2.4. Analysis of the Osteogenic Effect

#### 2.4.1. Cone-Beam Computed Tomography (CBCT) Examination

The samples were scanned using a CBCT (Meyer, SS-X9010DPro-3DE, China), with 10 *μ*m spot size and 55 kVp maximum voltage to determine the amount of newly formed bone. 3D images were acquired.

#### 2.4.2. Hematoxylin and Eosin (He) Staining

The specimens were cut and decalcified in 10% EDTA for one week after CBCT examination, dehydrated in 75% to 100% ascending alcohol concentrations, and then embedded in paraffin. Histological sections were prepared for HE analysis. Samples were then observed and photographed under a high-resolution microscope (Olympus, BX53, Japan).

## 3. Results

### 3.1. Clinical Evaluation

No animals died during the experiment, and sutures healed well. When operation sites were reexposed after sacrifice, both groups showed new bone formation at the defects on each side.

### 3.2. CBCT Analysis

**Figure 2:**
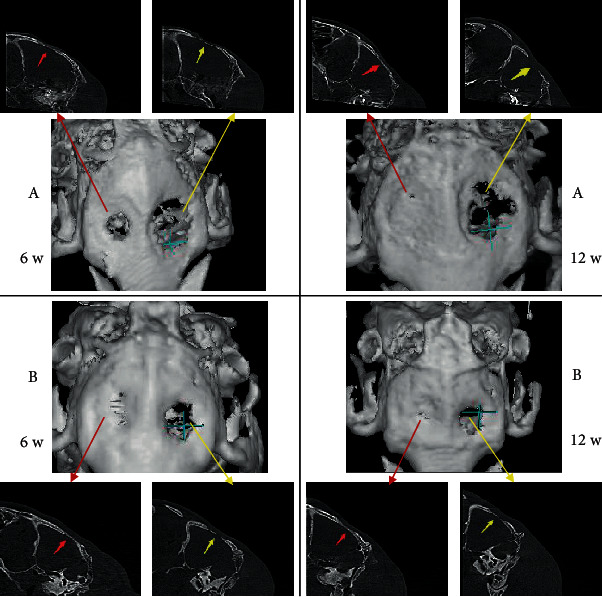
Images of newly formed bone 6 and 12 weeks after operation reconstructed by CBCT in two groups: (a) HA (red marrow) and blank (yellow marrow); (b) HA+CGF (red marrow) and CGF (yellow marrow).

CBCT results showed that the bone density of the right defects of all animals, both in Group A and Group B, 6 and 12 weeks after the operation, was much higher than that of the left defects. After 12 weeks in Group A (Figure 2(a), 12 w), the density of the right defects was lower compared to 6 weeks (Figure 2(a), 6 w), but the morphology and structures were more similar to the normal bone. The left defects remained thin and discontinuous, both 6 and 12 weeks after. In Group B, the changes in the right defects were similar to that in Group A, but the density reduction was much less, and the bone structure was more continuous and regular (Figure 2(b), 12 w). In the left defects (Figure 2(b), 12 w), a continuous lining structure was observed. However, in the 3D images, an irregular osteoid deposit was observed at the edge, while it remained discontinuous in the center (Figure 2(b), 12 w).

### 3.3. He Staining Analysis

**Figure 3:**
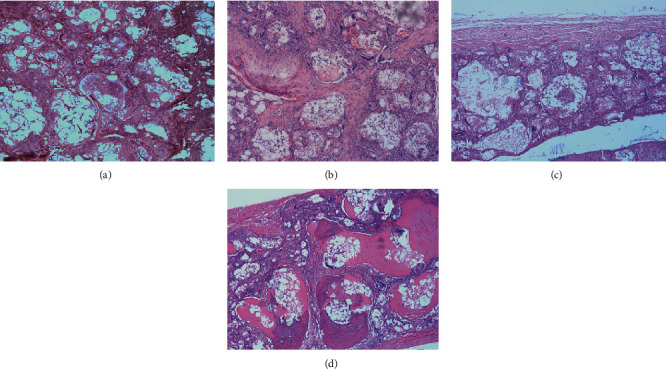
The microscopic histological images of cranial tissue sections (with HA or HA+CGF) of the two groups stained with HE, 6 and 12 weeks after operation: (a) HA 6 weeks; (b) HA+CGF 6 weeks; (c) HA 12 weeks; (d) HA+CGF 12 weeks.

**Figure 4:**
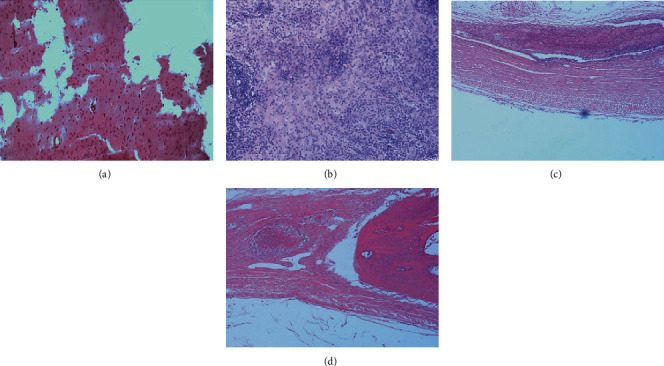
The microscopic histological images of cranial tissue sections (blank or with CGF) of the two groups stained with HE, 6 and 12 weeks after operation: (a) blank 6 weeks; (b) CGF 6 weeks; (c) blank 12 weeks; (d) CGF 12 weeks.

At the sixth week, in the right defect area (with HA or CGF+HA, Figures 3(a) and 3(b)) of both groups, fibrous tissue formed a crisscross structure around the bone meal particles, and a small amount of new bone, stained with a light powder, appeared. Some monocytes and multinuclear macrophages were observed, without lymphocytes. In the right defect area of Group B (Figure 3(b)), the fibrous tissue between the particles was thicker, which might be more suitable for the differentiation of blood vessels to provide more blood supply essential for the formation of the bone tissue. In the left defect area (blank or with CGF, Figures 4(a) and 4(b)), a large number of cells were observed, and no new bone tissue was seen.

At the twelfth week, there was continuous cortical bone around the right defect area (with HA or HA+CGF, Figures 3(c) and 3(d)) in the two groups. The cancellous bone, bone marrow, and connective tissue were observed in the center, and a large number of bone-like deposits generated around the HA powder particles. However, the more powdered new bone was observed in the right defect area (HA+CGF, Figure 3(d)) in Group B, which was thicker and more regular than that in Group A (Figure 3(c)), consistent with the results of CBCT. In the left defect area (without HA) of both groups (Figures 4(c) and 4(d)), there was no bone tissue formation, and there was no significant difference.

## 4. Discussion

The bone defect is quite common in the dental clinic. Tooth implantation [12] requires enough amount of surrounding bone. Horizontal and vertical bone absorption appear in periodontitis. Different sizes of maxillofacial cysts may also lead to bone loss. All of the above situations always require bone reconstruction. n-Hydroxyapatite has been widely studied over the years. Though many kinds of hydroxyapatite materials are used in the clinic, the results are not always ideal. The insufficient quantity and strength of newly formed bone remain unsolved in the clinic, and the cost is always high. The n-hydroxyapatite used in this research was newly obtained from Ostrea cucullate, a kind of shellfish that can be easily acquired. It is obtained via natural calcination. Its inorganic composition is very similar to the bone, has good biocompatibility, and a natural porous structure. It is a potential scaffold material that can be used in bone tissue engineering.

In our research, n-hydroxyapatite was used as a scaffold material in defect reconstruction. As our research progressed, defects were partly refilled with newly formed bone tissue. HE staining showed that more osteoblasts and osteoclasts appeared around the granules in the early stage, thus allowing a more active reconstruction. At a later stage, granules acted as bone formation centers, and a newly formed bone could be observed around them. Furthermore, osteocytes surrounded by mature bone tissues could be observed, indicating that the reconstruction was not only structural but also functional, which was rarely seen in the controls.

As a new generation of blood extract, CGF contains a high concentration of some growth factors [13], such as transforming growth factor (TGF), platelet growth factor (PDGF), and vascular endothelial growth factor (VEGF) [14]. They are vital to the formation of the bone tissue.

Hideo Masuki's study [14] showed that the levels of TGF-*β*1, PDGF-BB, IGF-1, and VEGF in CGF were significantly higher than those in PRP. Polypeptide growth factors are known to play an essential role in the growth and differentiation of cells involved in wound healing. Unlike PRP, CGFs do not dissolve rapidly following application. Instead, the strong fibrin gel in the matrix is slowly remodeled, similar to a natural blood clot. Thus, CGF prolongs the duration of growth factor activity, which is suitable for growth factor synergy, enhancing cell proliferation and osteogenic differentiation. Some studies in vitro showed that CGF cocultured with periodontal ligament fibroblasts, and bone marrow stromal stem cells induce differentiation into osteoblasts [11, 15, 16]. In our study, no sufficient amount of new bone tissue was observed in the bone defect area filled with CGF alone, and there was no significant difference between CGF and the blank group. The possible reason is that the proliferation and differentiation of osteoblasts need complex microenvironment and are regulated by many kinds of growth factors and pathways. Although the action of CGF takes longer than that of other blood extracts, it is still relatively short in the long process of osteogenesis. Another possible reason is that the limited bone defect area is large, and osteoblasts cannot be supported by the scaffold materials. The lack of a stable environment for bone differentiation leads to a small amount of bone formation in the central area of the defect, which is only covered by fibrous tissue. Studies [17] showed that CGF significantly promotes the proliferation of stem cells and has a dose-dependent effect. In a conditioned medium, CGF significantly accelerates osteogenesis of stem cells. Thus, we observed that the bone formation in defects filled with n-hydroxyapatite and CGF was greater than others.

In this study, CGF and n-hydroxyapatite combination had the best osteogenic effect, which was superior to other groups regarding the amount of osteogenesis and reconstruction of the bone structure. This may be related to the following: n-hydroxyapatite provides a reliable scaffold for the attachment of early osteoblasts; CGF can release growth factors in the early postoperative period and promote the differentiation and proliferation of osteoblasts [16]. In the middle and later stages, CGF gel was absorbed into the bone particles, which resulted in more vascular tissue formation, enhanced local vascularization, increased nutrient supply, and growth factor regulation during bone repair and regeneration, resulting in more bone tissue formation. This was also consistent with the results observed in HE stained sections. However, the specific mechanism needs further study. Therefore, we believe that CGF can significantly promote the repair of bone defects when combined with appropriate scaffolds, but the effect of CGF alone on a broad range of bone defects is not ideal. Studies [17] show that CGF can promote osteogenic differentiation in a time- and dose-dependent manner. Therefore, we also believe that if the growth factors in the blood can be concentrated and extracted using more advanced technology and combined with the corresponding scaffold materials, it will be more effective in the repair of bone tissue defects.

Here, we showed that the hydroxyapatite material extracted from oyster shell shows good biocompatibility. In the process of bone defect repair, it can be used as a stable scaffold material and has a particular bone induction effect, which provides an experimental basis and theoretical basis for its further clinical application. CGF contains a variety of growth factors, which can promote bone formation and vascularization. Its combined effect with hydroxyapatite material in bone defect repair is noticeable, but the specific mechanism needs further study. Moreover, CGF application alone in bone defect repair also needs further observation.

## Data Availability

The data used to support the findings of this study are available from the corresponding author upon request.

## Additional Points

*Additional Comments*. We choose CBCT as the imaging detection method but micro-CT, it is the limitation of this study.
